# *N*-Glycan on the Non-Consensus N-X-C Glycosylation Site Impacts Activity, Stability, and Localization of the Sd^a^ Synthase B4GALNT2

**DOI:** 10.3390/ijms24044139

**Published:** 2023-02-18

**Authors:** Virginie Cogez, Dorothée Vicogne, Céline Schulz, Lucie Portier, Giulia Venturi, Jérôme de Ruyck, Mathieu Decloquement, Marc F. Lensink, Guillaume Brysbaert, Fabio Dall’Olio, Sophie Groux-Degroote, Anne Harduin-Lepers

**Affiliations:** 1 CNRS, UMR 8576-UGSF-Unité de Glycobiologie Structurale et Fonctionnelle, Université de Lille, F-59000 Lille, France; 2 Department of Medical and Surgical Sciences (DIMEC), University of Bologna, General Pathology Building, Via San Giacomo 14, 40126 Bologna, Italy

**Keywords:** B4GALNT2, dimer, glycosyltransferase, *N*-glycan, unusual N-X-C glycosylation site

## Abstract

The Sd^a^ carbohydrate epitope and its biosynthetic B4GALNT2 enzyme are expressed in the healthy colon and down-regulated to variable extents in colon cancer. The human *B4GALNT2* gene drives the expression of a long and a short protein isoform (LF-B4GALNT2 and SF-B4GALNT2) sharing identical transmembrane and luminal domains. Both isoforms are trans-Golgi proteins and the LF-B4GALNT2 also localizes to post-Golgi vesicles thanks to its extended cytoplasmic tail. Control mechanisms underpinning Sd^a^ and B4GALNT2 expression in the gastrointestinal tract are complex and not fully understood. This study reveals the existence of two unusual *N*-glycosylation sites in B4GALNT2 luminal domain. The first atypical N-X-C site is evolutionarily conserved and occupied by a complex-type *N*-glycan. We explored the influence of this *N*-glycan using site-directed mutagenesis and showed that each mutant had a slightly decreased expression level, impaired stability, and reduced enzyme activity. Furthermore, we observed that the mutant SF-B4GALNT2 was partially mislocalized in the endoplasmic reticulum, whereas the mutant LF-B4GALNT2 was still localized in the Golgi and post-Golgi vesicles. Lastly, we showed that the formation of homodimers was drastically impaired in the two mutated isoforms. An AlphaFold2 model of the LF-B4GALNT2 dimer with an *N*-glycan on each monomer corroborated these findings and suggested that *N*-glycosylation of each B4GALNT2 isoform controlled their biological activity.

## 1. Introduction

**Figure 1:**
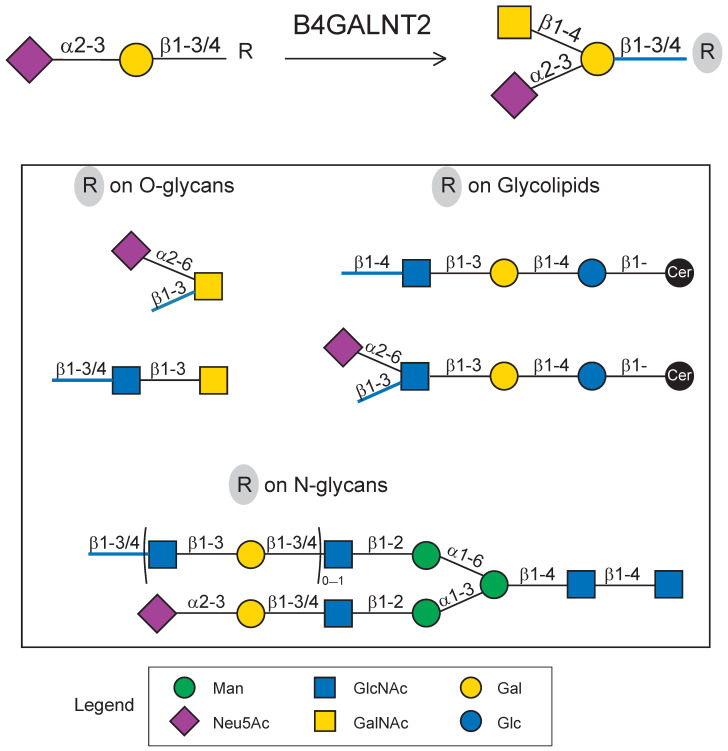
Schematic illustrating Sd^a^ biosynthesis. B4GALNT2 catalyzes the transfer of a *N*-acetylgalactosamine (GalNAc) residue onto sialylated disaccharides (Neu5Acα2,3Galβ1,3/4-) found either on *O*-glycans, glycolipids, or *N*-glycans. The possible R moieties (circled in grey) are represented in the box below the reaction. Glycans are drawn according to the Symbol Nomenclature for Glycans (SNFG) guidelines [17] with Drawglycan-SNFG [18], as indicated in the legend. The non-glycan ceramide moiety is represented here by a black-filled circle.

The Sd^a^ carbohydrate epitope (GalNAcβ1-4[Neu5Acα2-3]Galβ1-) is a histo-blood group antigen found on the erythrocytes of 90% of Caucasians [1,2] defining the blood group Sid [3,4]. It is also described in human adult tissues on *O*- and *N*-glycans of proteins and on glycolipids (Figure 1), as reviewed recently [3,5]. In particular, Sd^a^ carried by mucins *O*-glycans is a major structural feature in the mucus layer of the healthy human descending colon that disappears in cancer colon to the benefit of the sialyl Lewis x epitope (sLex, Neu5Acα2-3Galβ1-4[Fucα1-3]GlcNAc) [3,6]. It is also a tumor-associated carbohydrate antigen (TACA) found on glycolipids and glycoproteins in the prostate cancer renal cell carcinoma cell lines known as RM2 epitope [7,8]. A wide range of effects are attributed to Sd^a^ according to its environment, as reviewed in [5]. Among these, Sd^a^ inhibits metastasis in gastro-intestinal cancer cells and the stemness-associated malignant phenotype in cancerous colon cells [5,9,10], prevents the development of muscular dystrophy at the neuro-muscular junction [11,12], regulates clearance of the Von Willebrand factor [13], modulates the lytic function of cytotoxic T lymphocytes [14], impacts gut microbiota in mice [15], and regulates embryo attachment in a mouse model [16].

The Sd^a^/Cad synthase (i.e., β1,4-*N*-acetylgalactosaminyltransferase II, β1,4-GalNAcT-II, B4GALNT2) catalyzing the transfer of an *N*-acetylgalactosamine residue from UDP-GalNAc to the sialylated disaccharide Neu5Acα2,3Galβ1-R (Figure 1) is encoded by the *B4GALNT2* gene. The human gene maps to chromosome 17 and contains 11 exons [19,20]. *B4GALNT2* drives the expression of two major transcripts differing in their 5′ ends as a result of the use of two alternative first exons named exon 1S and exon 1L [19,20]. It was shown that in the gastrointestinal tract that exon 1S is predominantly used [6], and expression of this transcript requires ETS1 combined with DMTF1 and to a lesser extent SP1, both transcription factors (TF). However, these TF are not sufficient to explain the tissue-specific expression of B4GALNT2, suggesting the existence of additional regulatory elements [3,21,22]. The two transcripts were shown to encode two Sd^a^/Cad synthase protein isoforms with different-length cytoplasmic tails, which are designated short form (SF) and long form (LF) [6]. Both isoforms stably expressed in colon cancer cells LS174T lead to the formation of Sd^a^ antigen [23]. Recent studies have shown that the two isoforms have a Golgi localization and that the unusual, extended cytoplasmic tail of LF-isoform conferred an additional subcellular location of LF-B4GALNT2 in dynamic post-Golgi vesicles [24].

Although substantial achievements have been made towards elucidating the molecular mechanisms underpinning Sd^a^ expression, biosynthesis of this carbohydrate epitope and regulation of its expression in the human tissues appear to be complex and remain poorly understood [3]. Asparagine-linked glycosylation (*N*-glycosylation) of proteins is a widely distributed post-translational modification (PTM) of proteins in eukaryotic cells. It is a process initiated in the endoplasmic reticulum (ER) on the nascent proteins that occurs predominantly at the sequons N-X-T/S, where T = Thr = threonine, S = Ser = serine, and X can be any amino acid residue except proline (Pro, P), which is also known as classical or canonical motif N-[!P]-S/T-[!P]. *N*-glycosylation is a dynamic process playing roles in protein folding, maturation, secretion, and intracellular trafficking and also in disease progression [25]. Most glycosyltransferases involved in glycans construction of glycoproteins and glycolipids are themselves glycoproteins. These past years, several studies showed that elimination of their *N*-glycans could affect their activity, as recently reviewed [26]. In contrast to the mouse B4GALNT2 protein [27], sequence analysis of the two human B4GALNT2 polypeptides using the sequence-based predictor NetNGlyc [28] failed to predict the potential *N*-glycosylation site [20]. In addition, the human paralogue B4GALNT1 (also known as GM2/GD2 synthase) of B4GALNT2 in the CAZy family GT12 was shown to possess three *N*-glycosylation sites, and their removal resulted in a significant decrease in the enzyme activity [29]. Although 96.5% of *N*-glycosylation sites of mammalian proteins are found at a canonical sequon, minor and less predictable *N*-glycosylation sites such as N-X-C (1.3%), N-G (0.5%,) or N-X-V (0.4%) are possibly used; they are referred to as unusual or atypical glycosylation sites [30,31,32,33].

In this study, we reveal the existence of two potential *N*-glycosylation-sites in the human B4GALNT2 sequence. We show that the unusual N-X-C site (site 1) in the stem region is highly conserved during evolution in both vertebrate B4GALNT1 and mammalian B4GALNT2. This site is occupied by a complex-type *N*-glycan on mouse, rat, and human colonic B4GALNT2 and on the two recombinant isoforms of human B4GALNT2 produced in HeLa cells. To clarify the role of this *N*-glycan on human B4GALNT2 isoform localization, dimerization, and activity, we generated mutant protein isoforms lacking this glycosylation site. We show that this *N*-glycan slightly affects enzyme activity for both isoforms. Furthermore, our data suggest that it is essential for homodimers formation and for maintaining active conformation of the enzyme, as corroborated with a model of the glycosylated B4GALNT2 obtained with Alphafold2.

## 2. Results

### 2.1. Vertebrate B4GALNT2 Sequences Contain Unusual and Conserved N-Glycosylation Site(s)

**Figure 2:**
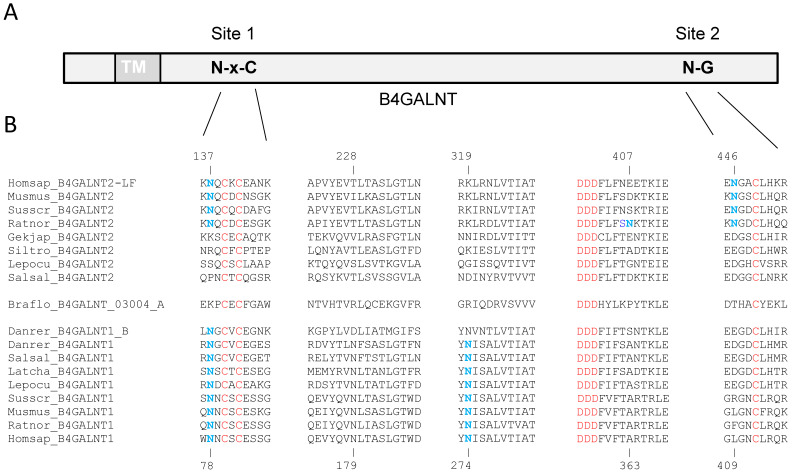
Conservation of the predicted *N*-glycosylation sites in vertebrates B4GALNT sequences of CAZy GT12. (**A**) Vertebrate B4GALNT sequences. Localization of the *N*-glycosylation sites N-X-C (site 1) and N-G (site 2) in vertebrate B4GALNT2 sequences are indicated in the schematic above. TM, transmembrane domain. Multiple sequence alignments were carried out with ClustalW using 136 full-length B4GALNT sequences (36 B4GALNT1, 70 B4GALNT2, and 20 B4GALNT1/2). (**B**) The MSA of B4GALNT sequences from the mammals *Mus musculus* B4GALNT2 (Musmus_B4GALNT2, BC139166) and B4GALNT1 (Musmus_B4GALNT1, NM_001478), *Rattus norvegicus* B4GALNT2 (Ratnor_B4GALNT2, XM_039087862) and B4GALNT1 (Ratnor_B4GALNT1, D17809), *Sus scrofa* B4GALNT2 (susscr_B4GALNT2, NM_001244330) and B4GALNT1 (Susscr_B4GALNT1, AK237665), the squamata *Gekko japanicus* B4GALNT2 (Gekjap_B4GALNT2, XM_01509212) and B4GLNT1 (Gekjap_B4GALT1, XM_015423486), the amphibian *Silurana tropicalis* B4GALNT2 (Siltro_B4GALNT2, XM_002935708) and B4GALNT1 (Siltro_B4GALNT1, XM_012956468), the non-teleostean fish *Lepisosteus oculatus* B4GALNT2 (Lepocu_B4GALNT2, XM_015362122) and B4GALNT1 (Lepocu_B4GALNT1, XM_006629420), the teleostean fish *Salmo salar* B4GALNT2 (Salsal_B4GALNT2, XM_01404566) and B4GALNT1 (Salsal_B4GALNT1, BT058789), and one invertebrate B4GALNT sequence of amphioxus *Branchiostoma floridae* (Braflo_B4GALNT,_03004_A, XM_002601302). Position of each predicted *N*-glycosylation site in the B4GALNT2 sequences is indicated above the alignment for the LF-B4GALNT2 sequence, and the conserved asparagine (N) appears in bold blue characters. In addition, the conserved *N*-glycosylation sites (N in blue) and positions described for the human B4GALNT1 sequence [29] are indicated below. Finally, the other conserved features of the B4GALNT sequences (i.e., cysteine residues (C) and D-X-D motif) are indicated in red characters.

No canonical *N*-glycosylation sites could be predicted using NetNGlyc in the human B4GALNT2 [20] or in the mouse B4GALNT2, whereas a typical sequon N-X-T/S could be predicted only in the rat B4GALNT2 sequence (Supplementary Figure S1). However, manual inspection of the human, mouse, and rat B4GALNT2 sequences suggested the existence of two unusual *N*-glycosylation sites N-X-C (site 1) and N-G (site 2), respectively, in the stem region and in the catalytic domain of the mammalian B4GALNT2 sequences. In the human B4GALNT2 isoforms, the sites 1 and 2 are positioned on N77 and N386 in the SF-B4GALNT2 and on N137 and N446 in the LF-B4GALNT2 (Figure 2).

Conservation of these predicted *N*-glycosylation sites in B4GALNT-related sequences was analyzed during vertebrate evolution. Firstly, we identified 187 B4GALNT-related sequences (61 B4GALNT1, 94 B4GALNT2, and 32 invertebrates B4GALNT1/2) in public databases at NCBI using a BLAST search approach and carried out multiple sequence alignments (MSA). The MSA of B4GALNT sequences from the mammals *Mus musculus*, *Rattus norvegicus*, *Sus scrofa*, the squamata *Gekko japanicus*, the amphibian *Silurana tropicalis*, the non-teleostean fish *Lepisosteus oculatus*, the teleostean fish *Salmo salar*, the amphioxus *Branchiostoma floridae*, and position of each predicted *N*-glycosylation site are shown in Figure 2. The predicted *N*-glycosylation sites in mammalian B4GALNT2 sequences were more or less conserved in B4GALNT2 sequences during vertebrate evolution and in the GM2 synthase (B4GALNT1). The first atypical *N*-glycosylation site N-X-C (site 1) is conserved in amniotes B4GALNT2 and in all B4GALNT1 sequences but not conserved in fish and frog B4GALNT2 sequences or in invertebrates B4GALNT1/2 sequences (Figure 2). The canonical sequon N-[!P]-S/T-[!P] found in the catalytic domain of rat B4GALNT2 sequences nearby the D-X-D motif is not conserved in the majority of vertebrate species or in B4GALNT1 sequences (Figure 2). The unusual *N*-glycosylation site N-G (site 2) is conserved in amniotes but lost in amphibian and fish B4GALNT2 sequences and is not conserved in B4GALNT1 sequences. Three *N*-glycosylation sites have been reported in the human B4GALNT1 sequence (N79, N179, and N274) [29]. The first site N79 described in this study is localized in a sequon (N-C-S) and conserved only in the mammalian B4GALNT1 sequences, whereas the two others are conserved in nearly all the vertebrate B4GALNT1 sequences (Figure 2).

### 2.2. N-Glycosylation of B4GALNT2 in Mammals

**Figure 3:**
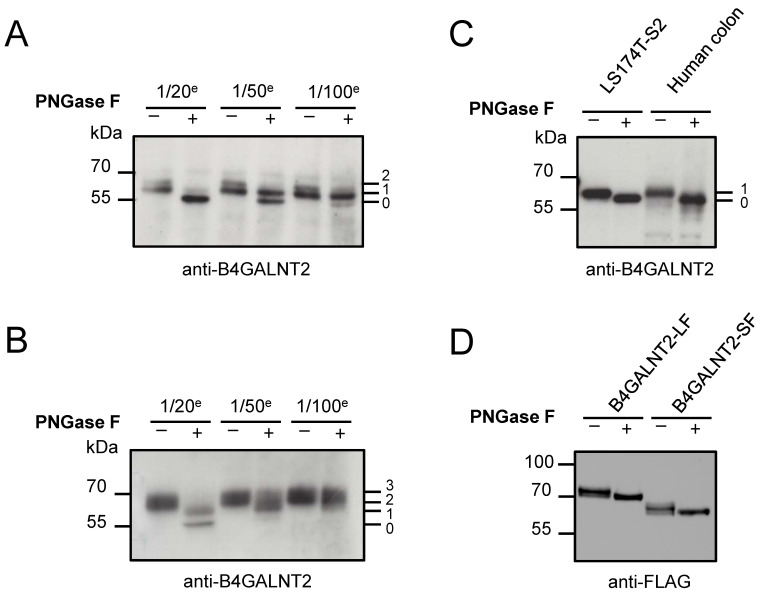
Partial PNGase F treatment on B4GALNT2. Serial dilutions (1/20^e^, 1/50^e^, or 1/100^e^) of PNGase F were used on mouse (**A**) or on rat (**B**) or on human (**C**) colon extracts. After SDS-PAGE and transfer on nitrocellulose membranes, immunoblotting with the anti-B4GALNT2 antibody reveals the presence in vivo of two, three, or one *N*-glycans on mouse, rat, and human B4GALNT2, respectively. PNGase F digestion of proteins from LS174T cells stably transfected with the SF-B4GALNT2 (LS174T-S2) and immunoblotting show a similar profile of glycosylation of the human B4GALNT2 (**C**). Finally, a representative immunoblot carried out with an anti-FLAG antibody shows a similar effect of PNGase F digestion on the LF-B4GALNT2 and SF-B4GALNT2 FLAG-tagged recombinant proteins transiently expressed in HeLa cells (**D**).

We next checked the occupancy of these predicted B4GALNT2 *N*-glycosylation sites in vivo in protein extracts from mouse, rat, and human colonic mucosa (Figure 3). We used serial dilutions of the endoglycosidic *N*-Glycosidase F (PNGase F) enzyme on colon protein extracts and ran SDS-PAGE. After transfer onto nitrocellulose membrane, the various glycosylated isoforms of the B4GALNT2 could be visualized with a specific anti-B4GALNT2 antibody directed towards their shared stem domain. As shown in Figure 3, three *N*-glycoforms (with no, one, or two *N*-glycans) of the B4GALNT2 were detected in mouse colon, supporting the existence of two *N*-glycans on the mouse enzyme in vivo (Figure 3A). Similarly, four *N*-glycoforms were observed on the rat B4GALNT2 (Figure 3B), supporting the presence of three *N*-glycans on the rat enzyme in colon. Only two *N*-glycoforms were observed on the SF-B4GALNT2 expressed in human colon and in the stably transfected LS174T-S2 cells [23] (Figure 3C), further suggesting the existence of a unique *N*-glycan on human B4GALNT2.

As a first step towards deciphering the role of this *N*-glycan, we constructed FLAG-tagged, full-length SF-B4GALNT2 and LF-B4GALNT2 in the p3×FLAG-CMV-10 expression vector, and recombinant proteins were expressed in HeLa cells. Partial digestion with PNGase F and Western blotting (WB) with an anti-FLAG antibody indicated the presence of one *N*-glycan on each recombinant protein, as shown previously in vivo (Figure 3D). Additional digestion assays of these recombinant proteins with Endo H indicated each recombinant B4GALNT2 isoform carried both Endo H-sensitive *N*-glycans (i.e., high-mannose/hybrid type) as found in ER and PNGase F-sensitive *N*-glycans (i.e., complex type) as found in the Golgi apparatus (Supplementary Figure S2). To determine which *N*-glycosylation site was occupied, C-terminus deletion constructs of the SF-B4GALNT2 (∆site 2 (∆262–506), ∆site 2 (∆97–506), and ∆site 1&2 (∆80–506)) were expressed in HeLa cells, and WB showed that the *N*-glycan is positioned on site 1 (Supplementary Figure S3).

### 2.3. Impact of N-Glycan on B4GALNT2 Activity and Sd^a^ Synthesis

**Figure 4:**
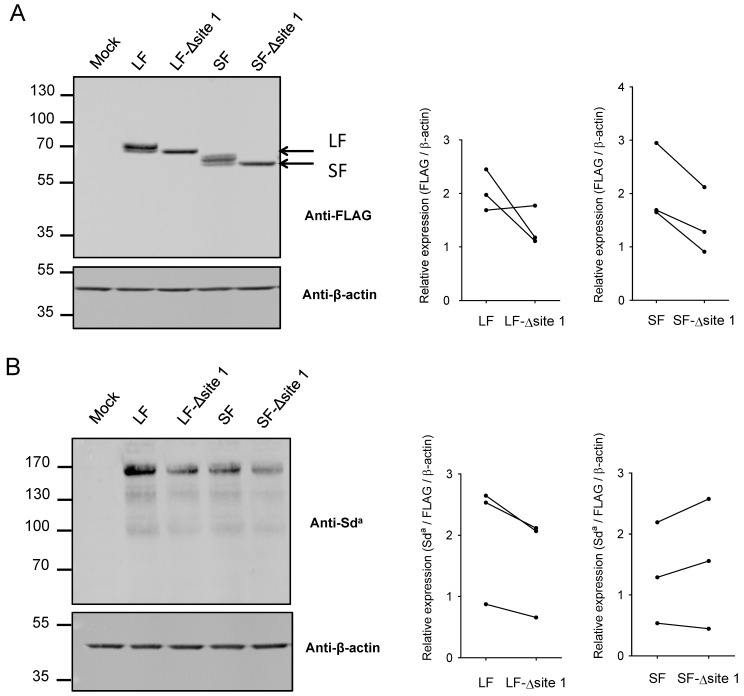
Effect of asparagine mutations (SF-∆site1 and LF-∆site 1) on B4GALNT2 production and activity in transfected HeLa cells. HeLa cells were transiently transfected with either an empty vector (Mock), the pFLAG-LF-B4GALNT2 (LF), and the pFLAG-SF-B4GALNT2 (SF) and their mutated counterparts pFLAG-LF-B4GALNT2-N137Q (LF-Δsite 1) and pFLAG-SF-B4GALNT2-N77Q (SF-Δsite 1). Total cell lysates were prepared and subjected to SDS-PAGE, and WB was performed with the anti-FLAG, anti-Sd^a^, and anti-β-actin antibody. Expression of the recombinant B4GALNT2 is shown 24 h after transfection (**A**). The Sd^a^ profile obtained after 24 h transfection is shown in (**B**). WB shown on the left side of the figure is one representative dataset of three distinct transfection experiments. ImageJ was used to compare the density of bands detected on WB. On the right side are represented quantifications of the expression level of B4GALNT2 isoforms normalized with β-actin and of Sd^a^ normalized relative to B4GALNT2/β-actin.

To gain insights into the role of this unique *N*-glycan in the human enzyme, we used site-directed mutagenesis substituting asparagine with glutamine residues N77Q in SF-B4GALNT2 and N137Q in LF-B4GALNT2, respectively, thereby eliminating the *N*-glycan on N-glycosylation site 1. Each construct was transiently transfected in HeLa cells in three individual experiments, and expression of each isoform was assessed by WB with the anti-FLAG antibody and normalized to β-actin. As illustrated in Figure 4A for one representative experiment (left panel), the absence of *N*-glycan slightly affected relative B4GALNT2 protein expression for both isoforms. Expression of the Sd^a^ antigen on glycoproteins in transfected HeLa cells was assessed by WB using the anti-Sd^a^ antibody KM694 (Figure 4B) and normalized to enzyme and β-actin expression. Sd^a^ expression was found to be slightly but not significantly reduced only in cells transfected with the mutated LF-B4GALNT2 form (Figure 4B).

**Figure 5:**
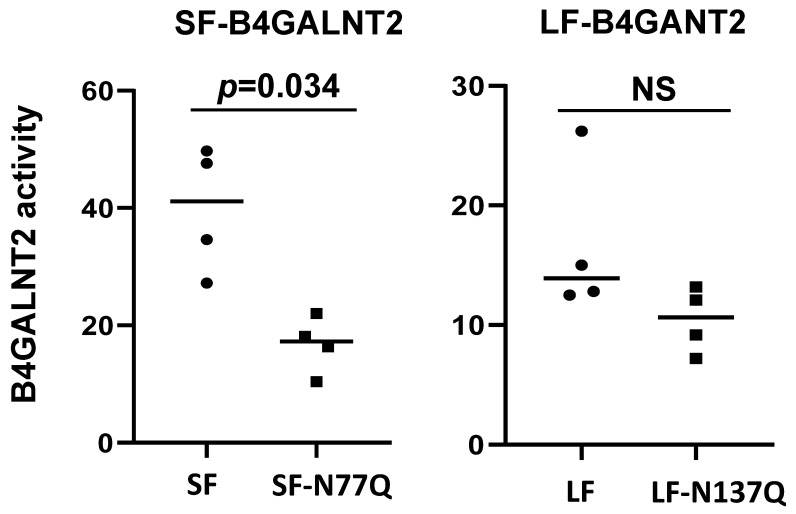
Normalized B4GALNT2 activity. COS-7 cells were transiently transfected with either an empty vector (Mock), the pFLAG-LF-B4GALNT2 (LF), and the pFLAG-SF-B4GALNT2 (SF) and their mutated counterparts pFLAG-LF-B4GALNT2-N137Q (LF-Δsite 1) and pFLAG-SF-B4GALNT2-N77Q (SF-Δsite 1). Total cell lysates were prepared and subjected to SDS-PAGE, and WB was performed with the anti-B4GALNT2 and β-actin antibody for quantification. Total cell lysates were used as an enzyme source for B4GALNT2 enzymatic assays using UDP-[^3^H]GalNAc and fetuin or asialofetuin as acceptors, as described previously [19]. The SF-Δsite 1 enzyme shows significantly reduced enzymatic activity compared to the SF isoform (**left side**), whereas the LF-Δsite 1 shows not significantly (NS) reduced activity compared to LF isoform (**right side**). Normalized data of B4GALNT2 activity in four experiments from three distinct transfections with statistical analysis (*t*-test for paired samples) are presented here; NS = not significant.

We also assessed the enzymatic activity of each full-length recombinant protein produced in COS-7 using in vitro assays. Interestingly, normalization of enzymatic activity for relative B4GALNT2 protein expression revealed a clear tendency to lower activity for mutant forms, which was more pronounced and statistically significant for the short form (Figure 5).

### 2.4. Impact of N-Glycan on Dimerization of B4GALNT2 Isoforms

**Figure 6:**
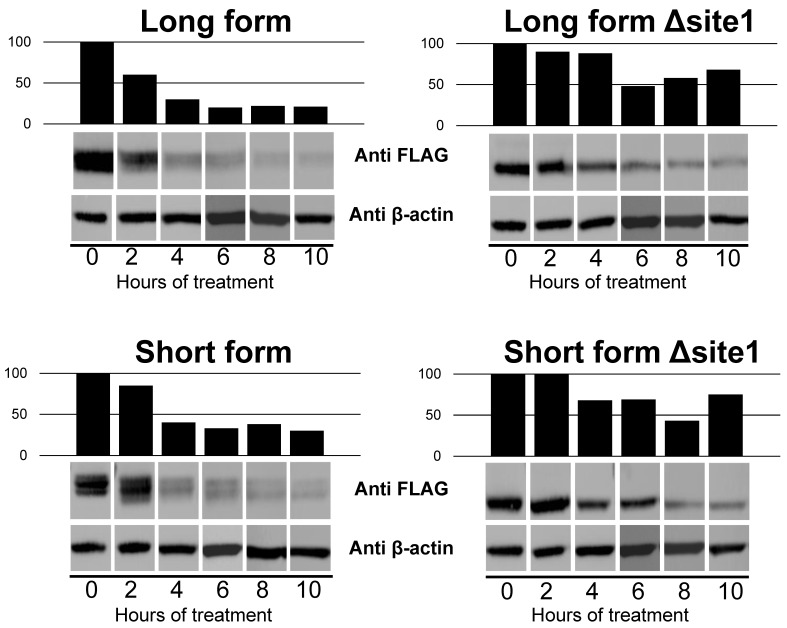
Effect of *N*-glycosylation site mutation on B4GALNT2 stability. HeLa cells were transiently transfected with pFLAG-LF-B4GALNT2 (LF), pFLAG-SF-B4GALNT2 (SF), and their mutated glycovariants pFLAG-LF-B4GALNT2-N137Q (LF-Δsite1) and pFLAG-SF-B4GALNT2-N77Q (SF-Δsite1). Twenty-four hours after transfection, cells were cultured in DMEM supplemented with 10% FBS and incubated with 5 μg/mL anisomycin for 2 to 10 h. After treatment, total cell lysates were prepared and subjected to SDS-PAGE, and WB was performed with the indicated antibodies. Graphs represent the intensity of the anti-FLAG signals normalized for the respective anti-β-actin signals. Statistical analysis by Student’s *t*-test for paired samples indicated that the intensity of the Δsite1 signals was statistically higher than that of the non-mutated form for both the long form (*p* = 0.0023) and the short form (*p* = 0.02).

We first assessed the impact of this unique *N*-glycan on protein stability. For that purpose, both the native SF-B4GALNT2 and LF-B4GALNT2 and their corresponding glycosylation mutants were expressed in HeLa cells in the presence of the protein synthesis inhibitor anisomycin for 2 to 10 h. As shown in Figure 6, our data indicated that *N*-glycosylation of the two B4GALNT2 isoforms had an impact on protein stability. For all time points, the mutated LF-B4GALNT2 and SF-B4GALNT2 forms were more expressed than their wild-type counterparts (*p* = 0.0023 and 0.02, respectively). These data strongly suggest that the *N*-glycan reduces the B4GALNT2 isoforms half-life. We also confirmed that with or without *N*-glycosylation, each B4GALNT2 isoforms transiently expressed in HeLa cells were not secreted (data not shown).

**Figure 7:**
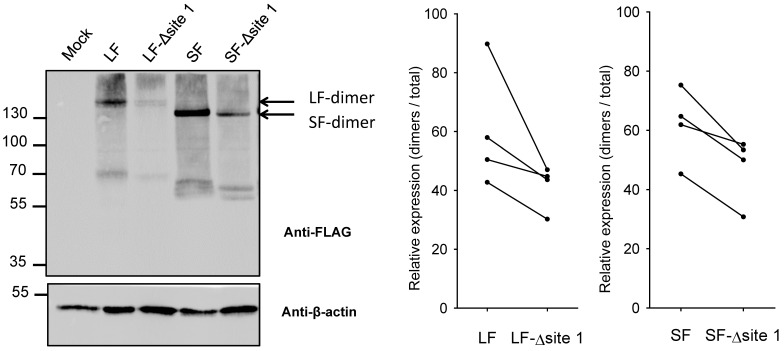
Effect of asparagine mutations (SF-∆site 1 and LF-∆site 1) on B4GALNT2 dimers formation. HeLa cells were transiently transfected with either an empty vector (Mock), the pFLAG-LF-B4GALNT2 (LF), and the pFLAG-SF-B4GALNT2 (SF) and their mutated counterparts pFLAG-LF-B4GALNT2-N137Q (LF-Δsite 1) and pFLAG-SF-B4GALNT2-N77Q (SF-Δsite 1). Total cell lysates were prepared and subjected to non-reducing SDS-PAGE, and WB was performed with the indicated antibodies. The figure shows a representative immunoblot of the B4GALNT2 dimers profile obtained after 24 h transfection. ImageJ was used to compare the density of bands detected on WB. On the right side are represented the relative expression level of B4GALNT2 isoforms dimers relative to dimers + monomers of B4GALNT2 isoforms/β-actin.

As glycosyltransferases potentially form enzymatically active homo- and heterodimers, we next investigated oligomerization of each B4GALNT2 glycovariant expressed in HeLa cells on non-reducing SDS-PAGE and WB. As illustrated in Figure 7 for one representative experiment (left panel), our data consistently show that eliminating the *N*-glycan decreased protein production and drastically impacted the relative formation of dimers for the LF-B4GALNT2 isoform and to a lesser extent for the SF-B4GALNT2 (Figure 7, right panel).

### 2.5. Subcellular Localization of B4GALNT2 Isoforms

**Figure 8:**
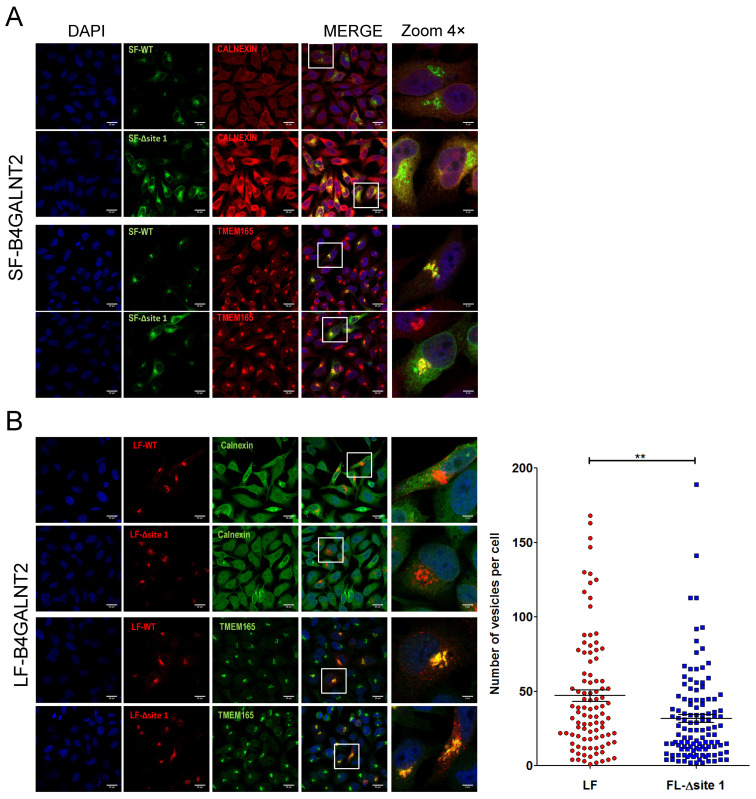
Subcellular localization of fluorescently labeled full-length B4GALNT2 glyco-variants in transfected HeLa cells using confocal microscopy. The SF-B4GALNT2 and its glyco-mutant SF-Δsite 1 were tagged with eGFP (green), and the LF-B4GALNT2 and its glyco-mutant LF-Δsite 1 were tagged with mCherry (red). Each tagged construct was transfected individually in HeLa cells. Their co-localization with ER and Golgi compartment markers Calnexin and TMEM165 (red in (A) and green in (B)) was examined by confocal microscopy. As previously reported [24], the SF-B4GALNT2 (**A**) and LF-B4GALNT2 (**B**) mostly co-localize with TMEM165 in the Golgi membranes, and LF-B4GALNT2 showed an additional localization in post-Golgi vesicles. The glyco-mutant SF-Δsite 1 (**A**) colocalized partially with Calnexin and TMEM165, whereas the LF-Δsite 1 (**B**) showed mostly the same subcellular localization as its non-mutated counterpart. The total number of red vesicles distinct from Golgi compartments was counted per cell and quantified using the ICY software (version ICY: 2.3.0.0, http://icy.bioimageanalysis.org, accessed on 5 November 2021), as previously described [24]. Data are plotted in whiskers plots, where means and SEMs are depicted as shown on the right side, panel B; they indicate a little but significant decrease in vesicles number in the LF-Δsite 1 transfected cells, as assessed by the Kruskal–Wallis non-parametric test with selected comparison using Dunn’s post hoc test. Scale bars: 5 µm; the white squares indicated the position of the 4× zoom image shown on the right side; ** represent the level of significance and correspond to *p* = 0.0019.

It was previously shown that the two B4GALNT2 isoforms localized in the Golgi and that LF-B4GALNT2 showed additional post-Golgi vesicle localization because of its extended cytoplasmic tail [24]. To assess the impact of *N*-glycan of B4GALNT2 onto its subcellular localization, we used fusion full-length B4GALNT2 C-terminal fluorescent-tagged proteins. The SF-B4GALNT2 glycovariants were coupled with green fluorescent protein (eGFP), while the LF-B4GALNT2 glycovariants were coupled with mCherry. We then performed immunostaining of HeLa cells overexpressing the various SF- and LF-B4GALNT2 fluorescent glycovariants with the Golgi and ER markers TMEM165 [34] and Calnexin, respectively, and analyzed subcellular localization of each protein using confocal microscopy (Figure 8). As previously reported, SF-B4GALNT2 co-localized with the Golgi marker TMEM165 [24]. By contrast, the SF-B4GALNT2 glyco-mutant displayed a characteristic pattern of cytoplasmic expression, which included but was not limited to Golgi. In fact, this pattern of expression largely colocalized with the ER marker Calnexin (Figure 8A). Interestingly, the expression of Calnexin, which is a molecular chaperone involved in the unfolded protein response, appeared to be increased in cells expressing this mutant. These data indicate that the absence of *N*-glycan profoundly alters the intracellular distribution of SF-B4GALNT2 and suggest that the accumulation of the unglycosylated form induced Calnexin overexpression. Surprisingly, the LF-B4GALNT2 glyco-mutant was mostly not affected by the absence of *N*-glycan, as it was found in the Golgi membranes and in post-Golgi vesicles, as described previously for LF-B4GALNT2 [24]. However, vesicles number counted per cell was significantly decreased in the non-glycosylated LF-B4GALNT2-transfected cells (Figure 8B).

### 2.6. Model of the B4GALNT2 Structure

**Figure 9:**
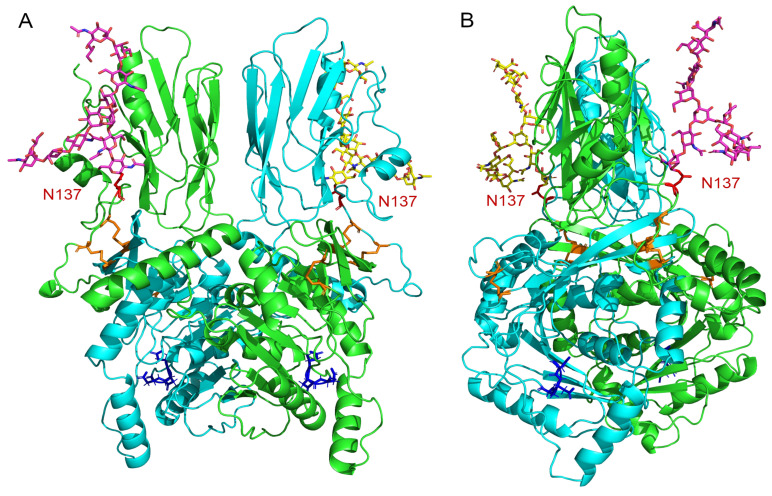
Three-dimensional model representation of B4GALNT2 dimer generated with AlphaFold2. (**A**) The two monomers are in green and cyan, and the *N*-glycosylation site 1 is depicted in red on both monomers, with a complex-type *N*-glycan depicted in yellow and pink (structure of the *N*-glycan described in Section 4); inter- and intra-monomeric disulfide bridges are colored orange, and the D-X-D motif in the catalytic cleft are shown in blue; (**B**) is a 90° rotation on the *y*-axis of (**A**).

To gain insights into the potential role of this *N*-glycan on B4GALNT2 dimerization from a 3D-structure representation of B4GALNT2, we built a model of the dimer exhibiting the *N*-glycan on site 1. For that purpose, we focused on the core protein shared by the LF- and SF-B4GALNT2 isoforms, removing the first 111 residues of LF-B4GALNT2 encompassing the cytoplasmic tail, a flexible domain with respect to the rest of the protein (see DynaMine backbone flexibility prediction of Supplementary Figure S4). Since this *N*-terminal part shows the worse confidence in the structural model of the AlphaFold database [35] for the *Homo sapiens* LF-B4GALNT2 (Supplementary Figure S4B), we built a model of the dimer with AlphaFold2 for the 112–566 sequence. We observed that the site 1 asparagine residues of both monomers are very accessible in the model: relative accessibility is 84.1% for chain A (ND2 accessibility = 58.311 Å²) and 82.6% for chain B (ND2 accessibility = 58.168 Å²), leaving a large space for a modification of this amino acid residue. We then further grafted a complex-type *N*-glycan on site 1 of both monomers (see Section 4 for details), which is shown in Figure 9. Even if the *N*-glycans are big, they are not long enough to block the access to the catalytic pocket. Interestingly, the asparagine residue bearing the *N*-glycan is located in a region that is not so flexible (Supplementary Figure S4) and is close to disulfide bridges, which limits the movements of the *N*-glycan to its own motions. Measurements in the structure show that the size of the *N*-glycan is inferior to 40 Å (~37 Å), while the distance between the N137 and the entry of the catalytic pocket is superior to 40 Å (~43 Å). Therefore, the two *N*-glycans in the dimer cannot sterically inhibit the enzymatic activity, which is in accordance with our experimental results.

## 3. Discussion

Bioinformatics approaches for *N*-glycosylation sites prediction are based on recognition of the sequon N-X-T/S. However, the presence of such a typical sequence is neither necessary nor sufficient for *N*-glycosylation. Although 96.5% of *N*-glycosylation sites of mammalian proteins are found at this canonical site, minor and less predictable N-glycosylation sites in glycoproteins such as cysteine-proximal acceptor sites N-X-C (1.3%) or N-G (0.5%) or N-X-V (0.4%) have been previously reported in a mouse *N*-glycoproteome study and are referred to as unusual glycosylation sites [33]. In this study, additional potential *N*-glycosylation sites in the mammalian B4GALNT2 sequences were identified that could not be predicted earlier [20,27]. Two sites, site 1 and site 2, were identified in the human SF- and LF-B4GALNT2 sequences and in the mouse B4GALNT2, and an additional typical *N*-glycosylation site was identified in the rat B4GALNT2 sequence (Figure 2). Analysis of the evolutionary conservation of these *N*-glycosylation sites in animal B4GALNT-related sequences (e.g., B4GALNT1 and B4GALNT2 sequences of the CAZy family GT12) revealed that the unusual site 1 was likely present in the B4GALNT ancestor since it is observed after vertebrate radiation in both B4GALNT1 and B4GALNT2 sequences. It was highly conserved in all the vertebrate B4GALNT1 sequences and was lost in the B4GALNT2 sequences in the fishes and amphibians branches during vertebrate evolution (Figure 2), further suggesting a solvent-accessible and functionally constrained site [36]. The unusual *N*-glycosylation site 2 N-G was not conserved in all B4GALNT-related sequences; in particular, it is absent in all the vertebrate B4GALNT1 sequences (Figure 2). This observation further suggested that this site was acquired in the common ancestor of mammals (Figure 2) and may have resulted in functional innovation for the mammalian B4GALNT2 since it was fixed during mammalian evolution. Located at the interface between catalytic and stem domains, this *N*-glycan could play an important role in the dimerization of B4GALNT2. Another *N*-glycosylation site was predicted in a canonical sequon N-[!P]-S/T-[!P] in the catalytic domain of the rat B4GALNT2, which is nearby the D-X-D motif (Figure 2). This site is not conserved in the majority of vertebrate species with the notable exception of ferret and elephant B4GALNT2 sequences (data not shown). Furthermore, it could not be predicted in primate, mouse, amphibian, or fish B4GALNT2 sequences, and this position is also not conserved in B4GALNT1 sequences (Figure 2). The gain of this site restricted to rat B4GALNT2 may affect the structure and molecular function of the rat enzyme, therefore conferring novel traits to this species.

A previous study reported the existence of three *N*-glycosylation sites in the human B4GALNT1 sequence (N79, N179, and N274) [29]. The first-described *N*-glycosylation site N79 is found in a typical sequon N-X-T/S and is conserved only in the mammalian B4GALNT1 sequences. It is juxtaposed to the highly conserved and unusual site 1 described in this study (N-N-C-T/S), where N78 could be *N*-glycosylated. The two other *N*-glycosylation sites are conserved in most vertebrate B4GALNT1 sequences but not in the B4GALNT2 sequences (Figure 2).

We then investigated whether these predicted *N*-glycosylation sites in human B4GALNT2 could be occupied in vivo. Protein extracts from human, mouse, and rat large intestine were treated with serial dilutions of PNGase F and run on SDS-PAGE. Immunoblotting with an anti-B4GALNT2 antibody revealed two, three, or four bands with a ~2.5 kDa molecular mass shift likely corresponding to the various glycoforms of B4GALNT2 in human, mouse, and rat colon, respectively (Figure 3). To determine whether all potential *N*-glycosylation sites could be used, various FLAG- and GFP-tagged recombinant proteins truncated or not in their catalytic domain were produced in HeLa cells. Our data demonstrated that the two human B4GALNT2 isoforms harbor a unique *N*-glycan located on the N-X-C site 1 (Figure 3 and Supplementary Figure S3). As frequently observed in other glycosyltransferases such as ST6GAL1 [37], the site 2 in human B4GALNT2 remained unglycosylated in vivo although the SF-B4GALNT2 isoform produced in HeLa cells sometimes shows little amounts of a second glycoform with two *N*-glycans (Figure 3, Figure 4 and Figure 7). Interestingly, the mouse B4GALNT2 that shares the same conserved *N*-glycosylation sites 1 and 2 appears to have a higher amount of the glycoform with two *N*-glycans in colon (Figure 3). In the mouse sequence, site 2 is a conventional sequon (N-G-S) and consequently likely occupied with an *N*-glycan compared to the rat sequence (N-G-D) or in the human sequence (N-G-A). Finally, further digestion assays using PNGase F and Endo H indicated the presence of a complex-type *N*-glycan on human B4GALNT2 site 1 (Figure 3 and Supplementary Figure S2).

It has long been known that *N*-glycosylation of proteins is a dynamic process involved in their folding, maturation, and trafficking in the secretory pathway [25]. Most of the resident Golgi glycosyltransferases involved in the last glycosylation steps of proteins and lipids are *N*-glycosylproteins themselves, and their *N*-glycan(s) are often associated with their enzymatic activity although the effects vary from protein to protein [26]. In the sialyltransferase family, for instance, the rat ST6GAL1 *N*-glycosylation on N146 and N158 is not an absolute requirement for α2,6-sialylation in vivo [37]. Similarly, for the human ST3GAL1, none of the *N*-glycans found on N27, N79, N114, N201, and N323 are essential to the enzymatic activity assayed in vitro [38,39]. However, the *N*-glycan found on the evolutionary conserved N211 glycosylation site of human ST3GAL2 dramatically affects in vitro activity of the enzyme, likely through influencing substrates recognition or enzyme folding [40]. In this study, we used biochemical methods and site-directed mutagenesis to evaluate the contribution of the *N*-glycan of the evolutionary conserved site 1 to the biological function of the two human B4GALNT2 isoforms. Elimination of this *N*-glycan in each isoform resulted in a moderate effect on the amount of each protein and Sd^a^ epitope formed, as assessed by WB and illustrated in Figure 4. However, normalized enzymatic activity assessed in a cell-free system was significantly lower for the unglycosylated short form (Figure 5). A similar tendency was observed, although it was less pronounced, for the long form (Figure 5).

To gain insights into the molecular mechanisms responsible for the decreased activity, we also investigated subcellular localizations, secretion, and potential dimerization of each B4GALNT2 isoform. Several studies have highlighted the crucial role of *N*-glycans in correct subcellular localizations and/or in facilitating and stabilizing proper protein folding [26], such as for the murine ST3GAL5 and chicken and murine ST8SIA1, which were unable to exit the ER once deglycosylated [41,42,43]. Interestingly, in contrast to the human B4GALNT1, whose intracellular localization was not affected by the absence of *N*-glycans [29], we showed in this study that the absence of the *N*-glycan on the SF-B4GALNT2 impaired its exit from the ER and Golgi localization (Figure 8A). Concomitantly, in cells expressing the mutated short form, we observed an overexpression of the molecular chaperone Calnexin, which could be indicative of the activation of the unfolded protein response. This possibility, which requires further investigations to be demonstrated, would indicate that without *N*-glycosylation, the short form fails to adopt the proper folding and to pass the cellular quality control mechanisms. On the other hand, the absence of the *N*-glycan on the LF-B4GALNT2 had almost no effect on its subcellular localization (Figure 8B). This later observation can be explained by the existence of a strong signal in the extended cytoplasmic tail driving Golgi targeting and post-Golgi sorting of the LF-B4GALNT2, which we previously described [3,24].

Most Golgi-glycosyltransferases are known to assemble into homodimers and heterodimers in vivo [44,45,46,47]. Previous protein chemistry experiments and mass spectrometry analysis have shown that all cysteine residues of the B4GALNT1 were involved in intra- and inter-disulfide bonds responsible for the formation of homodimers in an antiparallel orientation [48]. These cysteine positions are highly conserved in the human paralogue B4GALNT2 and are likely engaged in intra- and inter-disulfide bonds, fitting perfectly with the proposed dimer model (Figure 9). Because of the cysteine-rich context found for site 1, we also assessed dimer formation and the impact of *N*-glycan loss on the dimer formation. We showed that most of the full-length recombinant B4GALNT2 isoforms produced in HeLa cells are dimerized, whereas the lack of *N*-glycan at site 1 largely decreased homodimer formation (Figure 7). This data further suggested the crucial role of this *N*-glycan in dimerization, proper folding and enzyme activity of each B4GALNT2 isoform.

To gain insights into the structure/function relationships of B4GALNT2, we used Alphafold2 to produce a model of the LF-B4GALNT2 dimer deleted of the first 111 amino acid residues, and we grafted two *N*-glycans: one on site 1 of each monomer (Figure 9). The scores given by the AlphaFold2 modelling software (i.e., confidence score called ipTM + pTM) were very high, and the five models produced were highly homogeneous. However, the first-ranked model was slightly different, exhibiting only intra-monomeric disulfide bridges, while the four others showed inter- and intra-monomeric disulfide bridges, as experimentally shown for B4GALNT1 [48]. We selected one of the later for the addition of an *N*-glycan on the site 1 of each monomer, which is located in a region that is not flexible and readily accessible to the oligosaccharyltransferase (OST) for core *N*-glycan transfer (Supplementary Figure S4). As observed on this *N*-glycosylated B4GALNT2 dimer, the *N*-glycan, even if long and flexible, cannot physically block the substrate’s access to the catalytic pocket of the enzyme. In fact, the presence of the *N*-glycan not only does not hamper enzyme activity but, on the contrary, slightly increases it. It is interesting to put into perspective here the work of Gilmore’s laboratory showing that mammalian cells likely use two mechanisms for the *N*-glycosylation of proteins. The first one involves the OST complex SEC61-STT3A, which mediates co-translational glycosylation, while the second one involves the STT3B complex acting in a posttranslocational mode on a subset of cysteine-rich *N*-glycosylation sites skipped by STT3A [49,50]. In light of this work, dimerization of B4GALNT2 likely occurs concomitantly to *N*-glycosylation of site 1 in the ER. Our model shows that dimerization and *N*-glycosylation are both possible at the same time because no sterical hindrance can be observed, with the dimerization leaving space for the activity of the STT3B complex and vice versa.

Unlike the biosynthesis of DNA, RNA, and proteins, which are deterministic processes, glycosylation is intrinsically stochastic, being the product of the cooperative and competitive interaction of glycosyltransferases, glycosidases, and other enzymes organized along the secretory pathway. In this study highlighting the control of the glycosyltransferase B4GALNT2 by its own *N*-glycosylation on an atypical *N*-glycosylation site, we provide detailed evidence of how glycosylation controls itself, further enlightening the extreme complexity of the process.

## 4. Materials and Methods

### 4.1. Bioinformatic Analysis of B4GALNT Protein Sequences

The human B4GALNT1 (NM_001478) and B4GALNT2 (AJ517770 (SF) and AJ517771 (LF)) protein sequences were used as seed sequences for the identification of 187 B4GALNT homologues in the NCBI database using BLAST. B4GALNT sequences from the mammals *Mus musculus* (NM_001478 and BC139166), *Rattus norvegicus* (D17809 and XM_039087862), *Sus scrofa* (AK237665 and NM_001244330), the squamata *Gekko japanicus* (XM_015423486 and XM_01509212), the amphibian *Silurana tropicalis* (XM_012956468 and XM_002935708), the non-teleostean fish *Lepisosteus oculatus* (XM_006629420 and XM_015362122), the teleostean fish *Salmo salar* (BT058789 and XM_01404566), and the amphioxus *Branchiostoma floridae* (XM_002601302) were selected for multiple sequence alignment using Clustal W at PRABI and *N*-glycosylation sites prediction. The online predictors of *N*-glycosylation sites in proteins NetNGlyc was used (https://services.healthtech.dtu.dk/service.php?NetNGlyc-1.0, accessed on 29 April 2022) [51].

### 4.2. Antibodies and Reagents

Anisomycin, the polyclonal rabbit anti-B4GALNT2 antibody (HPA015721), the monoclonal mouse anti-GFP (G1546), the monoclonal mouse anti-Flag M2 (F3165), the polyclonal rabbit anti-TMEM165, and the mouse anti-β-actin antibodies were purchased from Sigma-Aldrich (Saint Quentin Fallavier, France). Anti-Sd^a^ (KM694) antibody was a kind gift from Dr. Taeko Dohi and Dr. Akiko Furuya (Biologics Research Laboratories, Research Division, Kyowa Hakko Kirin Co., Tokyo, Japan). The polyclonal goat anti-mouse horseradish peroxidase-conjugated IgG was purchased from Agilent Technologies (Santa Clara, CA, USA). The polyclonal goat anti-mouse horseradish peroxidase-conjugated IgM was purchased from Fisher Scientific (Illkirch, France). The polyclonal goat anti-rabbit horseradish peroxidase-conjugated IgG was purchased from Sigma-Aldrich (Saint Quentin Fallavier, France). The secondary antibodies Alexa fluor^®^ 488 anti-rabbit and Alexa fluor^®^ 568 anti-rabbit were from Life Technologies (Fisher Scientific, Illkirch Graffenstaden, France). The polyclonal rabbit anti-Calnexin was purchased from Enzo life technologies (Villeurbanne, France). The GFP expression vector pFx was a kind gift of Dr. Jack Röhrer (University of Zurich), the expression vectors pEGFP-N1 and pmCherry-N1 were from Clontech, whereas p3×FLAG-CMVTM-10 was from Sigma.

### 4.3. Plasmid Constructions of Human Long and Short B4GALNT2 Isoforms and Mutants

Full-length SF- and LF-B4GALNT2 cDNAs (AJ517770 and AJ517771) were obtained previously [20]. Full-length SF- and LF-B4GALNT2 were amplified by PCR using oligonucleotide primers containing *Not*I-*Xba*I restriction sites for directed ligation in a *Not*I-*Xba*I p3×FLAG-CMV10 digested vector (Supplementary Table S1). For the production of fluorescent chimera proteins, full-length SF- and LF-B4GALNT2 were amplified by PCR using oligonucleotide primers containing *Nhe*I-*Bgl*I or *Nhe*I-*BamH*I restriction sites, respectively, for directed ligation in *Nhe*I-*Bgl*I or *Nhe*I-*BamH*I pEGFP or pmCherry digested vectors (Table S1). The three truncated SF-B4GALNT2 constructs (∆site 1∆262–506, ∆site 2∆97–506, and ∆site 1&2∆80–506) deleted of the last 144, 409, and 426 amino acids in the catalytic domain were amplified by PCR using oligonucleotide primers containing *Nhe*I-*Bgl*II (SF-B4GALNT2∆262–506, SF-B4GALNT2∆97–506) or *Nhe*I-*BamH*I (SF-B4GALNT2∆80–506) restriction sites for directed ligation in a *Nhe*I-*Bgl*II or *Nhe*I-*BamH*I pFx or pYFP digested vector (Table S1), as previously reported [24].

The p.N77Q and p.N137Q mutations in the SF- and LF-B4GALNT2 were obtained as follows: Two PCR amplifications (PCR1 and PCR2) were carried out with couples of oligonucleotide primers, one of which was carrying the codon mutation (underlined), i.e., the forward primer GCCCGCCTGGCATTATGC and the reverse primer GCTTCACATTTGCACTGCTGTTTCGGGAACAGCCAG (PCR 1) and the forward primer CTGGCTGTTCCCGAAACAGCAGTGCAAATGTGAAGC and the reverse primer GCTTCTGGCTGTCATCAGCCAC (PCR2), shown in Table S1. The two amplicons from PCR1 and PCR2 were mixed and subjected to a third PCR with primers GCCCGCCTGGCATTATGC and GCTTCTGGCTGTCATCAGCCAC. The resulting cDNA fragment was digested by *Xho*I and *BspE*I present in the B4GALNT2 cDNA from either side of the mutation and introduced by fragment replacement in the p3×FLAG-CMV10 containing SF- or LF-B4GALNT2 digested with *Xho*I and *BspE*I. Similarly, these mutations (p.N77Q or p.N137Q) were introduced in the peGFP-SF-B4GALNT2 or in pmCherry-LF-B4GALNT2 constructs by fragment replacement using restriction sites *Hind*III and *EcoR*I.

### 4.4. Cell Culture and Transfections

HeLa cells (ATCC CCL-2, LGC Standards SARL, Molsheim, France) and COS-7 cells (ATCC CRL-1651) were routinely grown in Dulbecco’s modified eagle’s medium (DMEM) (Biowest, Nuaillé, France) supplemented with 10% fetal bovine serum (Biowest, Nuaillé, France) and maintained at 37 °C in a humidity-saturated 5% CO_2_ atmosphere.

The transfections of HeLa and COS-7 cells were performed using Lipofectamine 2000 transfection reagent (ThermoFisher Scientific, Waltham, MA, USA). Cells were grown in six-well plates until they reached 70–80% confluency, washed twice with UltraMEM (Lonza, Basel, Switzerland), and then transfected for 24 h with 2 µg of plasmid DNA and 4 µL of lipofectamine. After 4 h, the transfections were stopped by removal of the transfection mixture and the addition of fresh culture medium. When used, anisomycin (Sigma) was added 24 h after transfection for 2 to 10 h at the final concentration of 5 µg/mL.

The cell culture media were harvested 24 h post transfection. After methanol/chloroform precipitation, protein pellets were suspended in RIPA buffer (Tris/HCl 50 mM pH 7.9, NaCl 120 mM, NP 40 0.5%, EDTA 1 mM, Na3VO4 1 mM, NaF 5 mM) supplemented with a protease cocktail inhibitor (Roche Diagnostics, Penzberg, Germany). SDS-PAGE was performed on 4–12% Bis-Tris gels (Fisher Scientific, Illkirch, France), and the membrane was blotted using the polyclonal rabbit anti-B4GALNT2 antibody.

### 4.5. Western Blot Analysis

Cells were scraped in Dulbelcco’s Phosphate Buffer (DPBS, Lonza) and then centrifuged at 6000 rpm, 4 °C for 10 min. Supernatant was discarded, and cells were then suspended in RIPA buffer supplemented with a protease cocktail inhibitor, as described above. Cell lysis was performed by passing the cells several times through a syringe with a 26 G needle. Cells were centrifuged for 30 min, 4 °C, at 14,000 rpm. The protein concentration contained in the supernatant was estimated with the micro BCA Protein Assay Kit (Fisher Scientific, Waltham, MA, USA) according to the manufacturer’s instructions. Then, 10 µg of total protein lysate were mixed with NuPAGE Lithium Dodecyl Sulfate (LDS) Sample Buffer (Fisher Scientific, Waltham, MA, USA) pH 8.4 supplemented with 4% β-mercaptoethanol (Sigma-Aldrich, Saint Louis, MO, USA). Samples were heated 5 min at 95 °C and then separated on 4–12% Bis-Tris gels (Fisher Scientific, Waltham, USA) and transferred to nitrocellulose membrane Hybond ECL (GE Healthcare, Little Chalfont, UK). Membranes were blocked using TBS containing 0.05% Tween 20 (TBS-T) and 5% (*w*/*v*) non-fat dried milk or a Blocking Reagent (Sigma-Aldrich, Saint Louis, MO, USA) for at least 1 h at RT. Primary antibodies mouse anti-FLAG, mouse anti-β-actin, mouse anti-GFP, and mouse anti-Sd^a^ were incubated overnight at 4 °C in TBS-T and 5% (*w*/*v*) non-fat dried milk or TBS-T at respectively 1:1000, 1:10,000, 1:3000, and 1:500 dilution. After three TBS-T washes, membranes were then incubated with the appropriate peroxidase-conjugated secondary antibody (goat anti-mouse IgG from Agilent or goat anti-mouse IgM from Fisher Scientific, used at a dilution of 1:10,000 or 1:20,000 in TBS-T and 5% (*w*/*v*) non-fat dried milk or TBS-T) for 1 h at RT. After five TBS-T washes, blots were developed using enhanced chemiluminescence (West Pico Plus, ThermoScientific). The images were acquired using a CCD camera (Fusion Solo, Vilbert Lourmat, Collégien, France).

### 4.6. Total Proteins Preparation from Various Mammalian Gastrointestinal Tissues and Immunochemical Analyses

Total proteins extraction from frozen pieces of mouse or rat or human gastrointestinal tissues was achieved essentially as previously described [52] except for sodium deoxycholate. Protein concentration was determined with the Micro BCA Protein Assay Reagent kit (Biorad, Marnes-La-Coquette, France). First, 25 µg of total protein extract and 7 µg of controls LS 174T protein were boiled for 5 min for reducing Laemmli sample buffer and then resolved by SDS-PAGE on 4–12% minigels (Life Technologies, Fisher Scientific, Illkirch Graffenstaden, France). Proteins were transferred onto a nitrocellulose membrane (200 mA, 2 h).

For B4GALNT2 detection, a blocking step was performed using PBS/5% non-fat dried milk/0.05% Tween 20 overnight at 4 °C for subsequent incubation with 1:1000 of the anti-B4GALNT2 polyclonal antibody performed for 16 h at 4 °C in the same buffer. After 3 PBS/0.05% Tween 20 (PBS-T) washes, membranes were incubated with 1:10,000 horseradish peroxidase conjugated goat anti-rabbit antibody (Sigma Aldrich) in PBS-T and 5% (*w*/*v*) non-fat dried milk for 1 h at room temperature (RT). Membranes were washed three times for 10 min in PBS-T, and detection was achieved using enhanced chemiluminescence (SuperSignal West Femto Chemiluminescent Substrate, Amersham Biosciences, Little Chalfont, UK).

For β-actin detection, a blocking step was performed using PBS/5% non-fat dried milk/0.05% Tween 20 for 1 h at RT for subsequent incubation with 1:1000 of the monoclonal anti β-actin antibody (Sigma Aldrich). After three washing steps in PBS-T, membranes were incubated for 1 h at RT with 1:10,000 horseradish peroxidase conjugated goat anti-mouse antibody (Sigma Aldrich) in PBS-T and 5% (*w*/*v*) non-fat dried milk. Membranes were washed three times for 10 min in PBS-T, and detection was achieved using enhanced chemiluminescence (ECL 2 Western Blotting Substrate, Amersham Biosciences, Little Chalfont, UK).

### 4.7. PNGase F and Endo H Treatments

To examine the *N*-glycosylation modification, 50 or 10 µg of total protein extracts from frozen pieces of tissues or from transiently transfected cells, respectively, was mixed with 10× Glycoprotein Denaturing Buffer (New England Biolabs, Ipswich, MA, USA) and heated 10 min at 100 °C. Ten percent of NP 40 and 10× GlycoBuffer 2 (New England Biolabs, Ipswich, MA, USA) was added then completed with H_2_O and 500,000 U/mL of *N*-Glycosidase F (PNGase F) (New England Biolabs, Ipswich, MA, USA) or diluted PNGase F to make a 20 µL total reaction volume. Samples were incubated 1 h at 37 °C. Then, PNGase-F-treated and non-treated samples were dissolved in reducing NuPage LDS Sample Buffer and resolved by SDS-PAGE on 4–12% Bis-Tris gels (Fisher scientific, Illkirch, France). Detection of B4GALNT2 and β-actin was performed as previously described.

To examine *N*-glycan maturation, 10 µg of total protein lysate from transiently transfected HeLa cells were mixed with 10× Glycoprotein Denaturing Buffer (New England Biolabs, Ipswich, MA, USA) and heated 10 min at 100 °C. 10× GlycoBuffer 3 (New England Biolabs, Ipswich, MA, USA) was added and then completed with H_2_O and 500,000 U/mL of Endoglycosidase H (Endo H) (New England Biolabs, Ipswich, MA, USA) to make a 20 µL total reaction volume. Samples were incubated 1 h at 37 °C. Then, Endo-H-treated and non-treated samples were dissolved in reducing NuPage LDS Sample Buffer and resolved by SDS-PAGE on 4–12% Bis-Tris gels (Fisher Scientific, Illkirch, France). Detection of B4GALNT2 and β-actin was performed as previously described.

### 4.8. B4GALNT2 Enzyme Activity

COS-7 transfected with B4GALNT2 constructs as described above was harvested and homogenized in distilled water. B4GALNT2 activity of the homogenates was assessed as previously described [19] by the difference between incorporation of radioactive GalNAc on fetuin and asialofetuin.

Immunofluorescence localization of mutated B4GALNT2 was performed using confocal microscopy HeLa cells seeded on 12 mm round glass coverslips and grown for 24 h. Cells were then transfected with the different constructs tagged with eGFP or mCherry, as described in Table S1, using Lipofectamine 2000 (Thermo Fisher Scientific Bioscience). The immunostaining experiments were performed 24 h after transfection following a protocol described previously [24]. Briefly, after three washes with PBS, cells were fixed with paraformaldehyde solution at 3.6% in PBS for 20 min at RT, then permeabilized 10 min at RT in PBS containing 0.5% of Triton X-100. The saturation steps and incubation of antibodies were performed in blocking buffer (PBS containing 2% (*w*/*v*) BSA, 2% (*v*/*v*) FBS, and 0.2% (*w*/*v*) gelatin). Nuclei were stained with Dapi for 15 min in PBS before mounting coverslips with mowiol for observation using fluorescent confocal microscopy.

Different primary antibodies against markers of the subcellular compartments were used for the immunofluorescence assays: Anti-Calnexin (Enzo Life Sciences, New York, NY, USA) and anti-TMEM165 (Sigma) were, respectively, diluted at 1:100 and 1:300 in blocking buffer. The secondary antibodies, Alexa fluor^®^ 488 anti-rabbit and Alexa fluor^®^ 568 anti-rabbit, were diluted at 1:600 in blocking buffer. The subcellular localization of the different fluorescent proteins and the immunostaining were detected through an inverted Zeiss LSM700 (Oberkochen, Germany) confocal microscope with a 40× oil immersion objective. Data were collected using ZEN PRO 2.1 software (Zeiss) and analyzed with FIJI-WIN64 and ICY free software (Version 2.3.0.0). The vesicles were quantified using the ICY software (http://icy.bioimageanalysis.org, accessed on 5 November 2021) in the same manner as previously described in [24].

### 4.9. Flexibility Prediction

A flexibility prediction of the full-length LF-B4GALNT2 (isoform 1 of the Uniprot ID Q8NHY0) was performed with the DynaMine tool [53,54]. This tool gives a S² score that can be interpreted for flexibility prediction as flexible for S² < 0.69, rigid for S² > 0.8, and context-dependent in between.

### 4.10. Modeling of the B4GALNT2 Dimeric Structure

A model of the B4GALNT2 dimer was produced from the long protein isoform sequence between residues 112 to 566 (isoform 1 of the Uniprot ID Q8NHY0) with a locally installed version of the AlphaFold v2.1 software [55,56,57]. This version of AlphaFold allows building models of monomers as well as multimers. The options used were the following:

--is_prokaryote_list=false
--max_template_date=2021-11-17
--model_preset=multimer
--db_preset=full_dbs

Five models were generated with iptm+ptm scores between 0.90 and 0.92, which were all predicted as very good, and superimposing these models shows they are very similar. The first model was slightly different from the four others in that it did not exhibit the interchain disulfide bridges. Although the four models exhibited very similar predicted aligned error matrices, the third-ranked model with a very good matrix and a more homogeneous pLDDT graph compared to the second ranked model was chosen. This model was further used for the addition of a complex type *N*-glycan on the N137.

The accessibility (in Å²) of the two N137 of the dimeric model was computed with Naccess v2.1.1 [58]. The following complex-type *N*-glycan was added to the N137 residue of both monomers in the dimeric model: Neu5Acα2,3Galβ1,4GlcNAcβ1,2Manα1,3(Neu5Acα2,3Galβ1,4GlcNAcβ1,2Manα1,6)Manβ1,4GlcNAcβ1,4GlcNAcβ1-.

A 3D structure of the *N*-glycan was generated and minimized with the GLYCAM web carbohydrate builder [59]. The *N*-glycan was then manually grafted on the N137 of both monomers. A minimization of the model bearing the *N*-glycans was finally performed with YASARA [60].

The model was rendered with Pymol 2.3.0 [61], which was used for the production of all images of the structures of this manuscript.

### 4.11. Statistical Methodology

Data were plotted as scatter plots, and statistical analyses were performed using GRAPHPAD PRISM 5.0 software (GraphPad Software Inc., La Jolla, CA, USA) and were compared using a nonparametric Mann–Whitney test. Values were considered significantly different (*), with *p* < 0.05 (***: *p* < 0.001).

## Data Availability

Data are contained within the article or Supplementary Materials.

## Supplementary Materials

The following supporting information can be downloaded at: https://www.mdpi.com/article/10.3390/ijms24044139/s1.
