# Supplementary material for: N-Glycan on the Non-Consensus N-X-C Glycosylation Site Impacts Activity, Stability, and Localization of the Sda Synthase B4GALNT2
Source: Int J Mol Sci. 2023 Feb 18;24(4):4139. doi: 10.3390/ijms24044139 (PMC9959560; doi:10.3390/ijms24044139)
Supplement: Supplementary file 1 [file ijms-24-04139-s001.zip › ijms-2097724-supplementary.pdf]

N-glycan on the non-consensus N-X-C glycosylation site impacts activity, stability and localization of the Sd^a synthase B4GALNT2

Key words: B4GALNT2/dimer/glycosyltransferase/N-glycan/unusual NxC glycosylation site

Virginie Cogez[1], Dorothée Vicogne[1], Céline Schulz[1], Lucie Portier[1], Giulia Venturi[2], Jérôme de Ruyck[1], Mathieu Decloquement[1], Marc Lensink[1], Guillaume Brysbaert[1], Fabio Dall'Olio[2], Sophie Groux-Degroote[1], Anne Harduin-Lepers[1‡]

1 Université de Lille, CNRS, UMR 8576 - UGSF - Unité de Glycobiologie Structurale et Fonctionnelle, F-59000 Lille, France

2 Department of Experimental, Diagnostic and Specialty Medicine (DIMES), General Pathology Building, University of Bologna, Via San Giacomo 14, 40126 Bologna, Italy

[‡]**Corresponding author** : Anne Harduin-Lepers, Unité de Glycobiologie Structurale et Fonctionnelle, UMR CNRS 8576, Université de Lille, Faculté des Sciences et Technologies, 59655 Villeneuve d'Ascq, France. Phone: +33 320 33 62 46; Fax: +33 320 43 65 55; E-mail: anne.harduin-lepers@univ-lille.fr; orcid.org/ 0000-0002-1233-3799

Running title: B4GALNT2 *N*-glycosylation

Supplementary data: Figure S1, Figure S2, Figure S3, Figure S4 and Figure S5

Figure S1

NetNGlyc 1.0

https://services.healthtech.dtu.dk/service.php?NetNGlyc-1.0

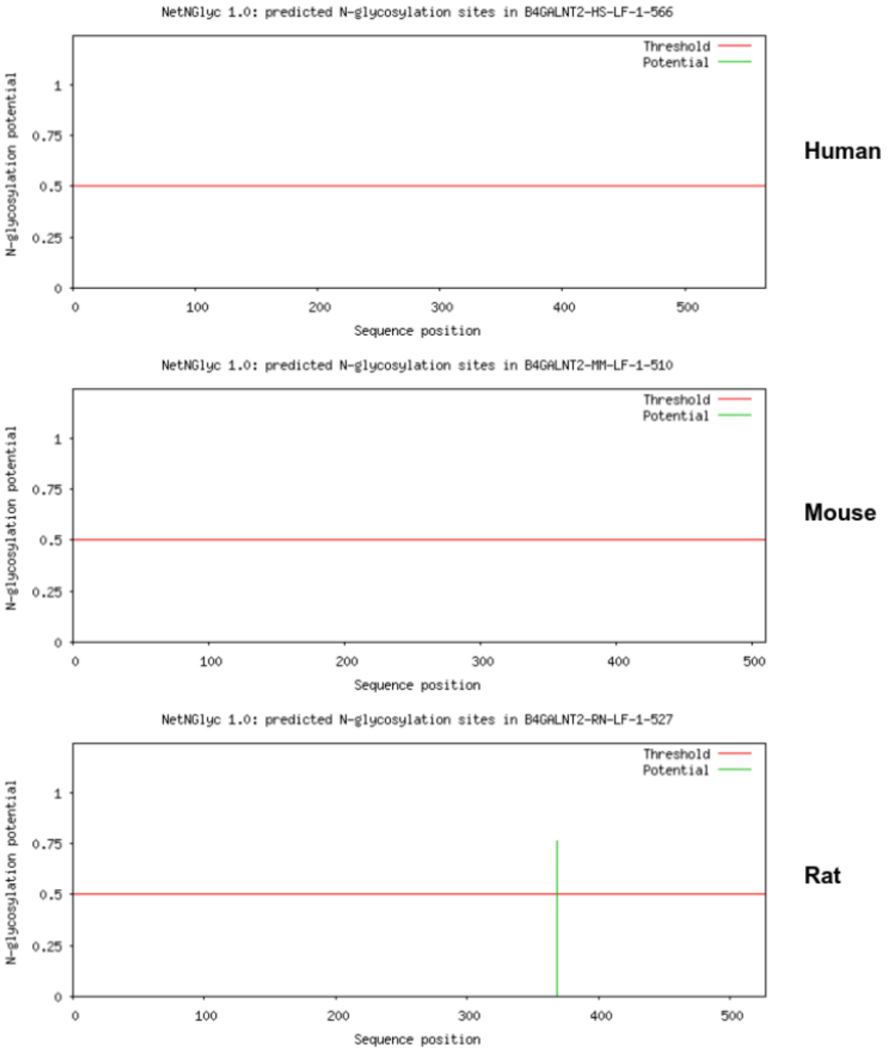

Site
TKYVLWVDDDFLFSNKTKIEVLVDVLE

Figure S1: N-glycosylation sites prediction. The B4GALNT2 protein sequences of human (LF-B4GALNT2), mouse and rat B4GALNT2 were selected for *N*-glycosylation sites prediction with the sequence-based predictor NetNGlyc (Gupta, R. and Brunak, S. 2001). Sequence analysis of the human and mouse B4GALNT2 polypeptides failed to predict potential *N*-glycosylation site, whereas only one site (N346) with a prediction score above 0.5 corresponding to a regular sequon could be predicted in the rat B4GALNT2 sequence. The rat *N*-glycosylation site is shown below the graphs.

Figure S2

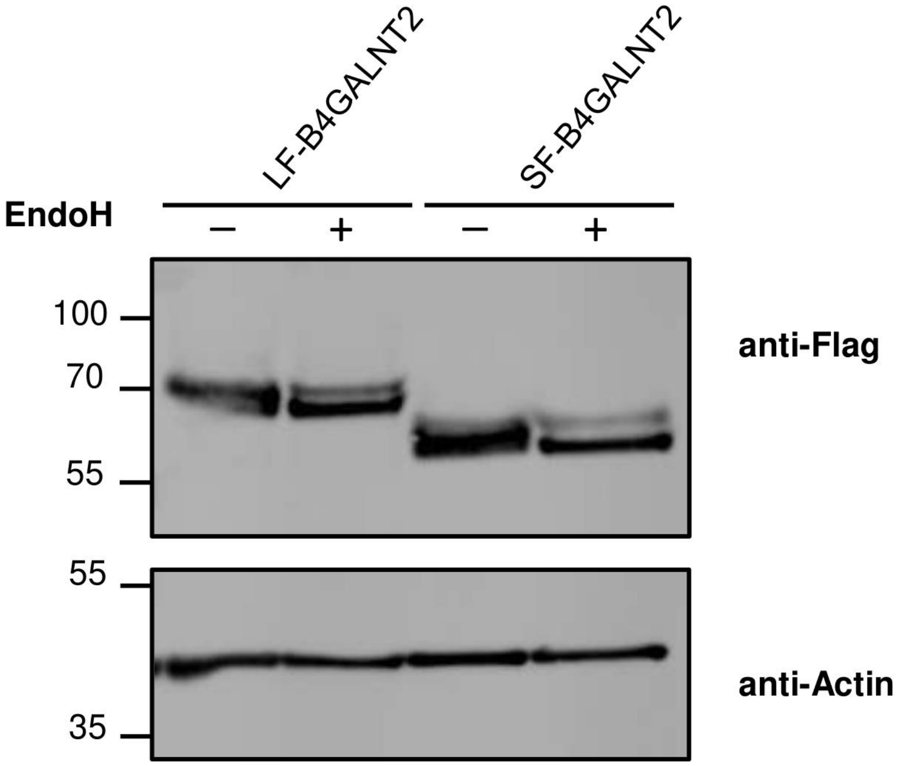

Figure S2 : Endoglycosidase H (Endo H) analysis of SF- and LF-B4GALNT2. The pFLAG-SF-B4GALNT2 and pFLAG-LF-B4GALNT2 were transiently transfected in HeLa cells. Twenty-four h post-transfection, cells extracts were digested 1 h at 37 °C with 500,000 U/ml of Endo H (+) or left untreated (-), subjected to SDS-PAGE and transferred on nitrocellulose membranes. Immunoblotting carried out with the anti-FLAG shows a partial digestion of SF-B4GALNT2 and LF-B4GALNT2 indicating the ongoing maturation and acquisition of complex-type N-glycan on the two protein isoforms.

Figure S3

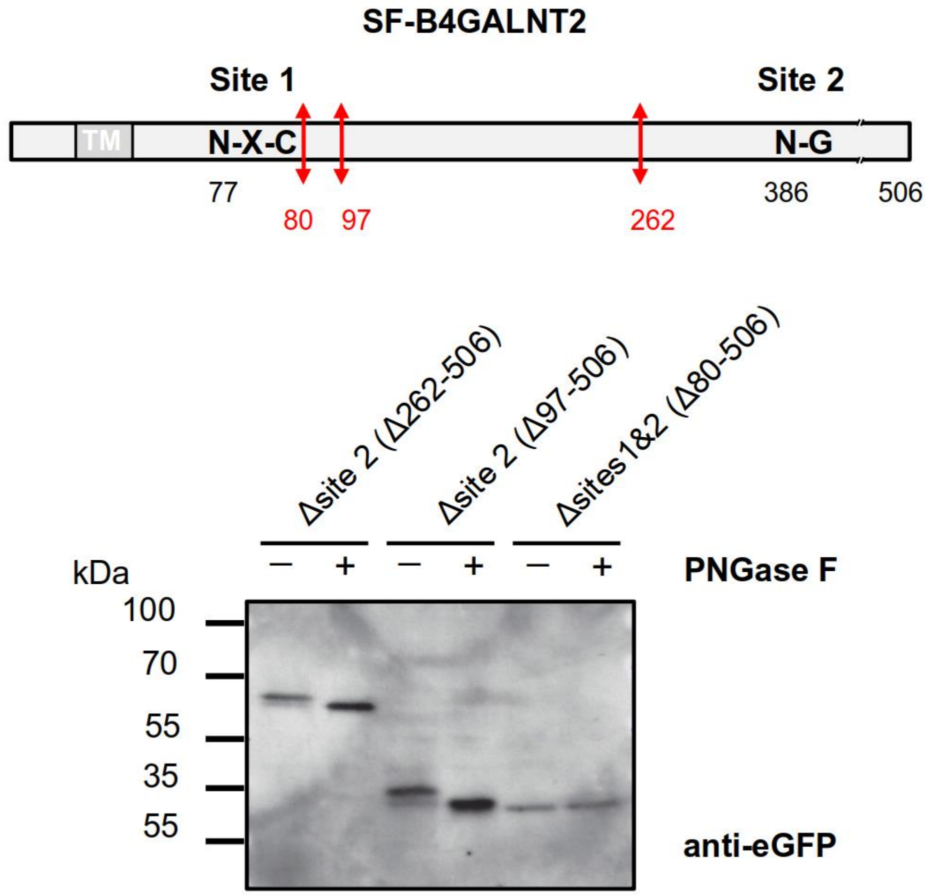

Figure S3: Identification of the occupied *N*-glycosylation site in human B4GALNT2. To identify which of the two predicted *N*-glycosylation sites (site 1 and site 2) illustrated on the top of the figure would be occupied in the human B4GALNT2, three constructs of the SF-B4GALNT2 (Δsite 2 Δ262-506, Δsite 2 Δ97-506 and Δsite 1&2 Δ80-506) deleted in the catalytic domain and including one or two *N*-glycosylation sites were made. HeLa cells were transiently transfected with these truncated isoforms coupled with green fluorescent protein (eGFP). Twenty-four h post-transfection, cells extracts were digested 1 h at 37 ℃ with PNGase F (+) or left untreated (-), subjected to SDS-PAGE and transferred on nitrocellulose membranes. Immunoblotting carried out with the anti-eGFP shows a shift in mobility of the Δsite 2 Δ262-506 and Δsite 2 Δ97-506 isoforms, but not of the Δsite 1&2 Δ80-506 further suggesting that site 1 is occupied with an *N*-glycan.

Figure S4

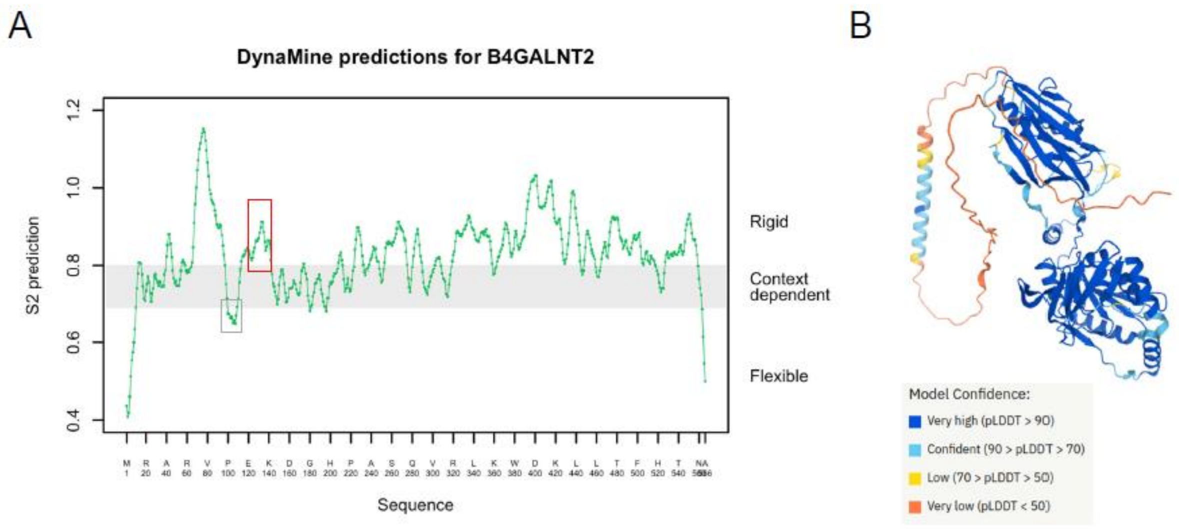

Figure S4: Flexibility prediction of B4GALNT2. (A) DynaMine flexibility prediction of the human LF-B4GALNT2 isoform; the region 100-110 (dashed grey square) is predicted flexible, which allows large movements of the upstream domain (stem region); the area around the *N*-glycosylation site (N137) is predicted rigid (red square); (B) AlphaFold model of the LF-B4GALNT2 from the AlphaFold database (https://alphafold.ebi.ac.uk/entry/Q8NHY0) highlighting the high confidence of the core structure and lower confidence of the flexible N-terminal region.

Table SI: Primers and plasmids used for B4GALNT2 glycovariants constructions. Name of the construct, sequence of the oligonucleotide primer and name of the expression vector are indicated in this Table. Restriction sites are underlined in the primer sequence.

Construct	Forward primer	Reverse primer	Vector	Ref
SF-B4GALNT2	TTTGCGGCCGCGACTTCGGGCGGCTCGAGATTTCTGTG	TTTTCTAGATTATGCGGCACATTGGAGATGGTTCTTG	pFLAG-CMV10	This study
LF-B4GALNT2	TTTGCGGCCGCGGGGAGCGCTGGCTTTTCCGTGGGA	TTTTCTAGATTATGCGGCACATTGGAGATGGTTCTTG	pFLAG-CMV10	This study
SF-B4GALNT2	CCTAGCTAGCGCCACCATGACTTCGGGCGGCTCG	GAAGATCTCTGCGGCACATTGGAGATG	pEGFP	This study
LF-B4GALNT2	CCTAGCTAGCGCCACCATGGGGAGCGCTGGCTTT	CGGGATCCCCTGCGGCACATTGGAGATG	pmCherry-N1	(Groux-Degroote, S., Schulz, C., et al. 2018)
SF-B4GALNT2Δ262-506	CCTAGCTAGCGCCACCATGACTTCGGGCGGCTCG	GAAGATCTCGTTTCTGAGCTTCCTCTC	pFx	This study
SF-B4GALNT2Δ97-506	CCTAGCTAGCGCCACCATGACTTCGGGCGGCTCG	GAAGATCTTATAGGCATCCTGAAAGTT	pFx	This study
SF-B4GALNT2Δ80-506	CCTAGCTAGCGCCACCATGACTTCGGGCGGCTCG	TTCAGGATCCCACTGATTTTTCGGGAACAGCC	pYFP	(Groux-Degroote, S., Schulz, C., et al. 2018)
PCR1	GCCCGCCTGGCATTATGC	GCTTCACATTTGCACTGCTGTTTCGGGAACAGCCAG	-	This study
PCR2	CTGGCTGTTCCCGAAACAGCAGTGCAAATGTGAAGC	GCTTCTGGCTGTCATCAGCCAC	-	This study
PCR3	GCCCGCCTGGCATTATGC	GCTTCTGGCTGTCATCAGCCAC	-	This study
